# Systemic lupus erythematosus and the risk of cardiovascular diseases: A two-sample Mendelian randomization study

**DOI:** 10.3389/fcvm.2022.896499

**Published:** 2022-09-02

**Authors:** Shuo Huang, Fugang Huang, Chunyun Mei, Fengyuan Tian, Yongsheng Fan, Jie Bao

**Affiliations:** ^1^The First School of Clinical Medicine, Zhejiang Chinese Medical University, Hangzhou, China; ^2^School of Basic Medical Sciences, Zhejiang Chinese Medical University, Hangzhou, China

**Keywords:** systemic lupus erythematosus, cardiovascular disease, coronary artery disease, myocardial infarction, atrial fibrillation, ischemic stroke, Mendelian randomization, single nucleotide polymorphism

## Abstract

**Background:**

Previous observational studies have suggested that the causal role of systemic lupus erythematosus (SLE) in the risk of cardiovascular diseases (CVDs) remained inconsistent. In this study, we aimed to investigate the causal relationship between SLE and CVDs by two-sample Mendelian randomization (MR) analysis.

**Methods:**

Genetic instruments for SLE were obtained from a public genome-wide association study (GWAS) with 4,036 patients with SLE and 6,959 controls. Summary statistical data for CVDs, including coronary artery disease (CAD), myocardial infarction (MI), atrial fibrillation (AF), ischemic stroke (IS), and its subtypes, were identified from other available GWAS meta-analyses. The inverse-variance weighted (IVW) method was used as the primary method to estimate the causal effect. The simple- and weighted-median method, MR-Egger method, and MR pleiotropy residual sum and outlier (MR-PRESSO) were provided as a supplement to the IVW method. Besides, we performed sensitivity analyses, including Cochran's Q test, MR-Egger intercept test, and leave-one-out analysis, to evaluate the robustness of the results.

**Results:**

A total of 15 single-nucleotide polymorphisms (SNPs) were identified after excluding linkage disequilibrium (LD) and potential confounding factors. According to the IVW results, our MR study indicated that genetically predicted SLE was not causally connected with the risk of CVDs [CAD: odds ratio (OR) = 1.005, 95% confidence interval (CI) = 0.986–1.024, *p*-value = 0.619; MI: OR = 1.002, 95% CI = 0.982–1.023, *p*-value = 0.854; AF: OR = 0.998, 95% CI = 0.982–1.014, *p*-value = 0.795; IS: OR = 1.006, 95% CI = 0.984–1.028, *p*-value = 0.621; cardioembolic stroke (CES): OR = 0.992, 95% CI = 0.949–1.036, *p*-value = 0.707; small vessel stroke (SVS): OR = 1.014, 95% CI = 0.964–1.067, *p*-value = 0.589; large artery stroke (LAS): OR = 1.030, 95% CI = 0.968–1.096, *p*-value = 0.352]. Analogical findings could be observed in supplementary MR methods. Sensitivity analyses suggested that the causal estimates were robust.

**Conclusion:**

Our two-sample MR analysis provided no evidence that genetically determined SLE was causally associated with the risk of CVDs.

## Introduction

Systemic lupus erythematosus (SLE) is one of the archetypal autoimmune diseases that can involve numerous organs or systems, including the skin, kidney, hematologic and nervous system, among others (1). The global prevalence of SLE ranged from 13 to 7,713.5 in every 100,000 individuals (2). In recent decades, with the improved awareness of SLE, comprehensive therapy, and prevention of complications, the overall survival rate has enhanced dramatically, with 5-, 10-, and 15-year survival rates of 96, 93, and 76%, respectively (3). However, the all-cause standardized mortality ratio for patients with SLE was still 2.6 times higher than that for the general population (4).

Several studies suggested that patients with SLE were more susceptible to cardiovascular diseases (CVDs), including coronary artery disease (CAD), myocardial infarction (MI), atrial fibrillation (AF), and stroke. A recent meta-analysis proclaimed that, compared with the unexposed cohort, the relative risk of patients with SLE suffering from CVDs was 1.98, especially lupus nephritis (5). Another retrospective cohort indicated that SLE could increase the incidence of CAD (OR 1.42, 95% CI 1.40–1.44) by enrolling 252,676 patients with SLE and 758,034 matched patients without SLE (6). In Korea, similar results for MI, AF, and stroke could be observed after an 8-year follow-up (7, 8). On the contrary, another meta-analysis and cohort study suggested that the incidence of CVDs did not differ significantly between patients with SLE and the control population (9, 10). Since the observational study was more likely prone to confounders, such as smoking, type 2 diabetes (T2D), and low-density lipoprotein (LDL), different studies were more likely to reach divergent conclusions. Therefore, it is necessary to further explore the causal association between SLE and CVDs.

Mendelian randomization (MR) is an epidemiological method that assesses causality between exposure and outcome depending on genetic variants (11). As genotypes are assigned randomly during meiosis and preceded phenotypes, it can address the influence of confounding factors and reverse causation (12). In recent years, MR study was widely applied to explore the causal association between multiple exposures and CVDs, such as sleep duration (13), alcohol intake (14), obesity (15), blood pressure (16), and major depressive disorder (17).

In this study, we aimed to estimate the potential causal relationship for SLE with the risk of CVDs that encompassed CAD, AF, MI, ischemic stroke (IS), and its subtypes by a two-sample MR study.

## Methods

### Study design

**Figure 1:**
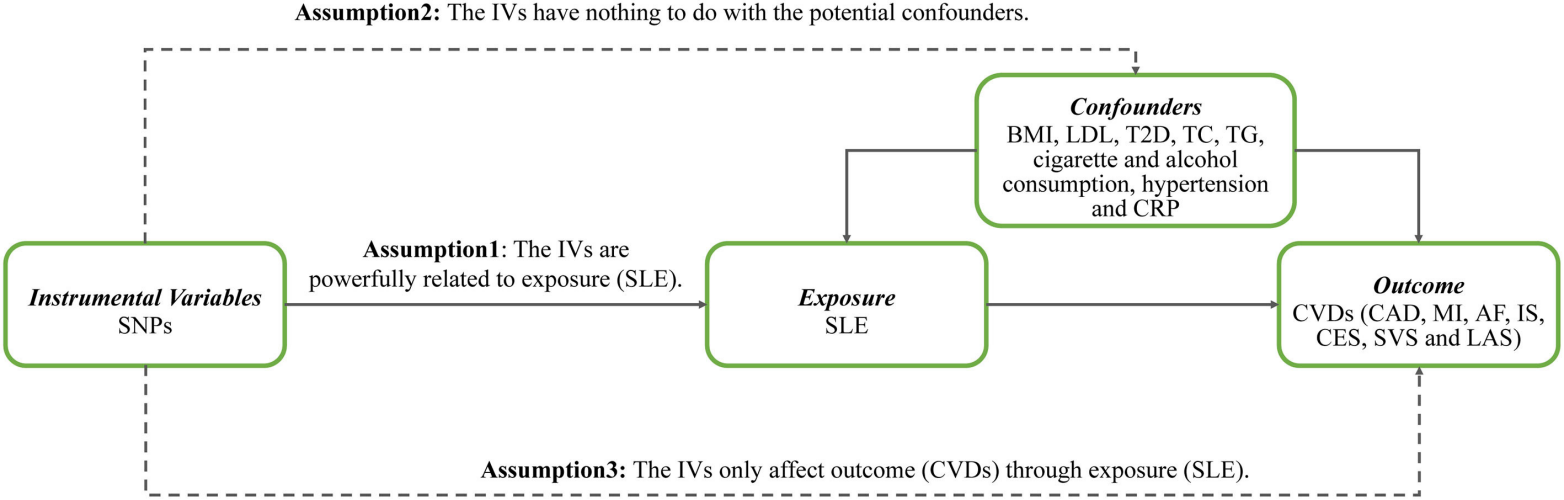
Three key assumptions of Mendelian randomization study. SNPs, single-nucleotide polymorphisms; IVs, instrumental variables; SLE, systemic lupus erythematosus; BMI, body mass index; LDL, low-density lipoprotein; T2D, type 2 diabetes; TC, total cholesterol; TG, triglyceride; CRP, C-reactive protein; CVDs, cardiovascular diseases; CAD, coronary artery disease; MI, myocardial infarction; AF, atrial fibrillation; IS, ischemic stroke; CES, cardioembolic stroke; SVS, small vessel stroke; LAS, large artery stroke.

The persuasive conclusion is obtained only when the following assumptions for the MR study are satisfied. First, the genetic instrumental variables (IVs) should be powerfully related to exposure (SLE). Second, the genetic IVs have nothing to do with the potential confounders, including body mass index (BMI), LDL, T2D, total cholesterol (TC), triglyceride (TG), hypertension, C-reactive protein (CRP), and others. Third, the genetic IVs only affect the outcome (CVDs) through SLE. Figure 1 shows three key assumptions of this MR study. Ethical approval of participants was not necessary as this research was based on the publicly available database.

### Data sources

Summary data on SLE were obtained from a meta-analysis of genome-wide association studies (GWASs) including 10,995 European participants with 4,036 cases and 6,959 controls (18). This study totally covered 644,674 single-nucleotide polymorphisms (SNPs), and all cases fulfilled the SLE diagnostic criteria of the American College of Rheumatology.

The GWAS meta-analysis with 48 studies was applied to extract the genetic variants associated with CAD and MI (19). This study assembled 60,801 cases (~70% cases had a reported history of MI) and 123,504 controls, nearly 77% individuals with European ancestry. All cases satisfied the CAD diagnosis, including MI, acute coronary syndrome, chronic stable angina, or coronary stenosis >50%. Atrial Fibrillation Genetics Consortium conducted a large-scale GWAS meta-analysis with 65,446 cases (84.2% European individuals) and 522,744 controls, reporting 12,149,979 SNPs (20). All cases suffered from paroxysmal, permanent AF, or atrial flutter. Genetic variants related to IS and its subtypes were based on another GWAS study from the MEGASTROKE consortium, covering ~8 million SNPs (21). According to the Trial of Org 10172 in Acute Stroke Treatment criteria, IS was further classified as cardioembolic stroke (CES) with 7,193 cases, small vessel stroke (SVS) with 5,386 cases, and large artery stroke (LAS) with 4,373 cases. Supplementary Table 1 describes the basic information of the above GWAS studies.

### SNPs selection

In the original study, Bentham et al. (18) identified 25 SNPs of genome-wide significance (*p*-value < 5 × 10^−8^). To ensure the independence of these 25 SNPs, we used LD-pruned (pairwise *r*^2^ < 0.001, window size = 10,000 kb) by the clump_data command using the R software (22). In addition, these SNPs were searched at the GWAS threshold (*p*-value < 5 × 10^−8^) by the PhenoScanner V2 database (http://www.phenoscanner.medschl.cam.ac.uk/) to rule out the influence of potential confounders (BMI, LDL, T2D, TC, TG, cigarette and alcohol consumption, hypertension, and CRP) (23). Furthermore, we calculated the *F*-statistic to assess the extent of weak instrument bias (24). The proportion of variance (*R*^2^), which was explained by these selected SNPs, was evaluated by the formula of 2 × MAF × (1 – MAF) × β^2^ (25). The smallest effect detected by the sample size of the outcome to provide 80% statistical power at an α level of 5% was calculated by using the online mRnd power tool [https://shiny.cnsgenomics.com/mRnd/ (26)].

### Statistical analysis

In this two-sample MR analysis, we used five methods [inverse-variance weighted (IVW), simple- and weighted-median, MR-Egger, and MR pleiotropy residual sum and outlier test (MR-PRESSO)] to derive the causal estimates between SLE and CVDs. The IVW analysis is the primary method in our MR study because it provides the most persuasive estimates when the directional pleiotropy of the IVs is absent (27, 28). The simple median yields causal effects, where <50% of information comes from valid IVs; the weighted median requires more than 50% of valid IVs (29). The MR-Egger method provides the causal estimates based on the slope from the weighted regression of the IVs-outcome relationship on the IVs-exposure relationship (27). The MR-PRESSO method detects horizontal pleiotropy and reappraises the causal effect after eliminating the pleiotropic IVs (30).

**Figure 2:**
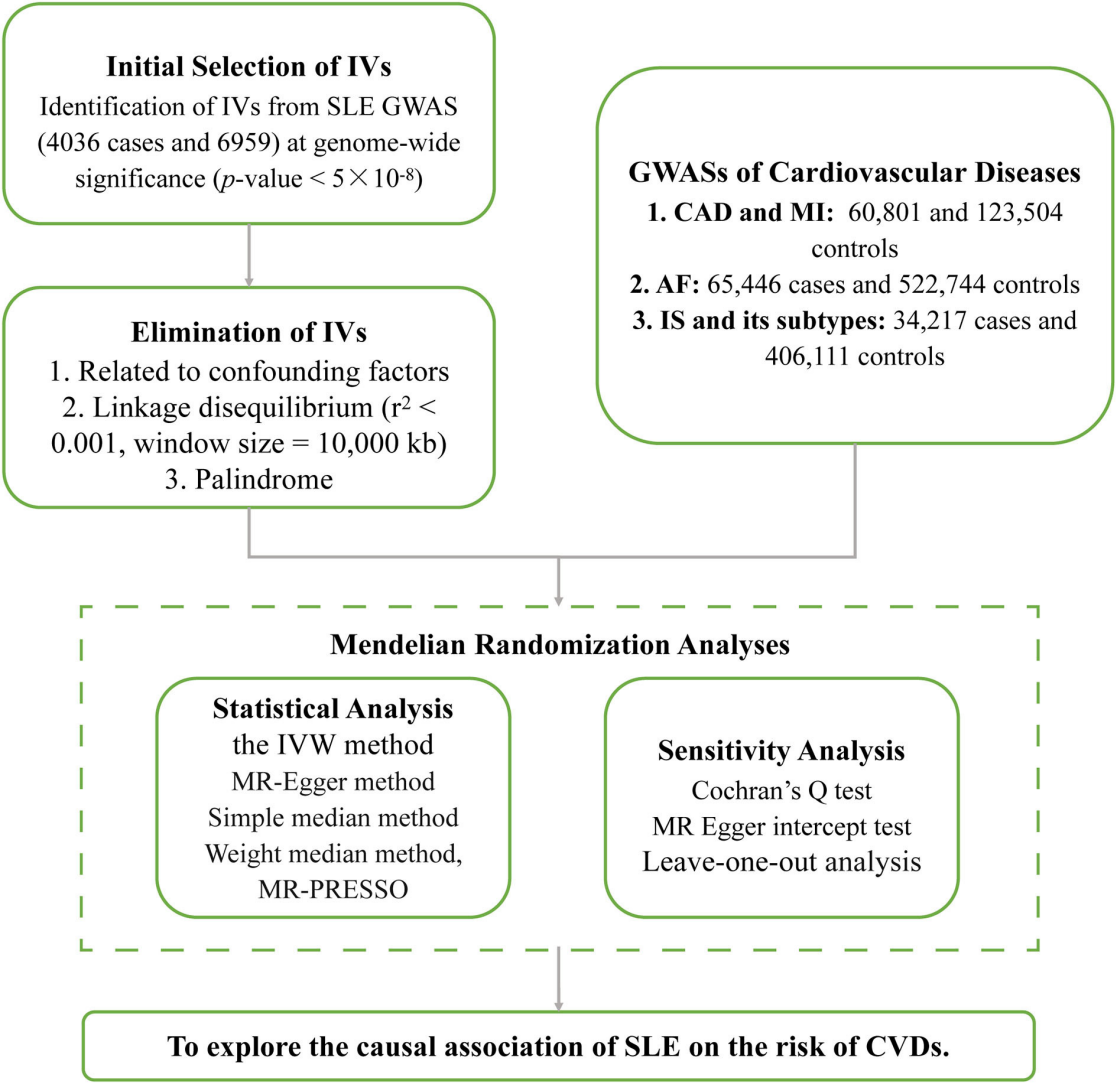
The flowchart of this Mendelian randomization study. IVs, instrumental variables; SLE, systemic lupus erythematosus; GWAS, genome-wide association study; CAD, coronary artery disease; MI, myocardial infarction; AF, atrial fibrillation; IS, ischemic stroke; IVW, inverse-variance weighted method; MR-PRESSO, Mendelian randomization pleiotropy residual sum and outlier test.

To further explain the potential pleiotropy, we conducted a series of sensitivity analyses. Cochran's *Q*-test quantifies the heterogeneity of IVs, with a value of *p* < 0.05 indicating heterogeneity (31). The deviation of the MR-Egger intercept from zero determines whether there exists a directional horizontal pleiotropy. Furthermore, we also used the leave-one-out analysis to assess whether the causal effect was disturbed by a single SNP (27). Figure 2 displays the flowchart of IVs selection and MR analyses.

All statistical analyses were performed by MR-PRESSO (1.0) (30) and TwoSampleMR (0.5.5) (22) packages using the R software (3.6.1), and a value of *p* < 0.05 was considered statistically significant if not otherwise stated.

## Result

On account of linkage disequilibrium (LD) and confounding factors (BMI, LDL, T2D, TC, TG, cigarette and alcohol consumption, hypertension, and CRP), 10 SNPs were excluded. Finally, a total of 15 SNPs were taken as effective IVs. The *F*-statistic of all selected SNPs was over 10, which ranged from 137.29 to 1,817.47, suggesting that there was no weak instrument bias. Supplementary Table 2 describes the detailed information about these SNPs.

**Figure 3:**
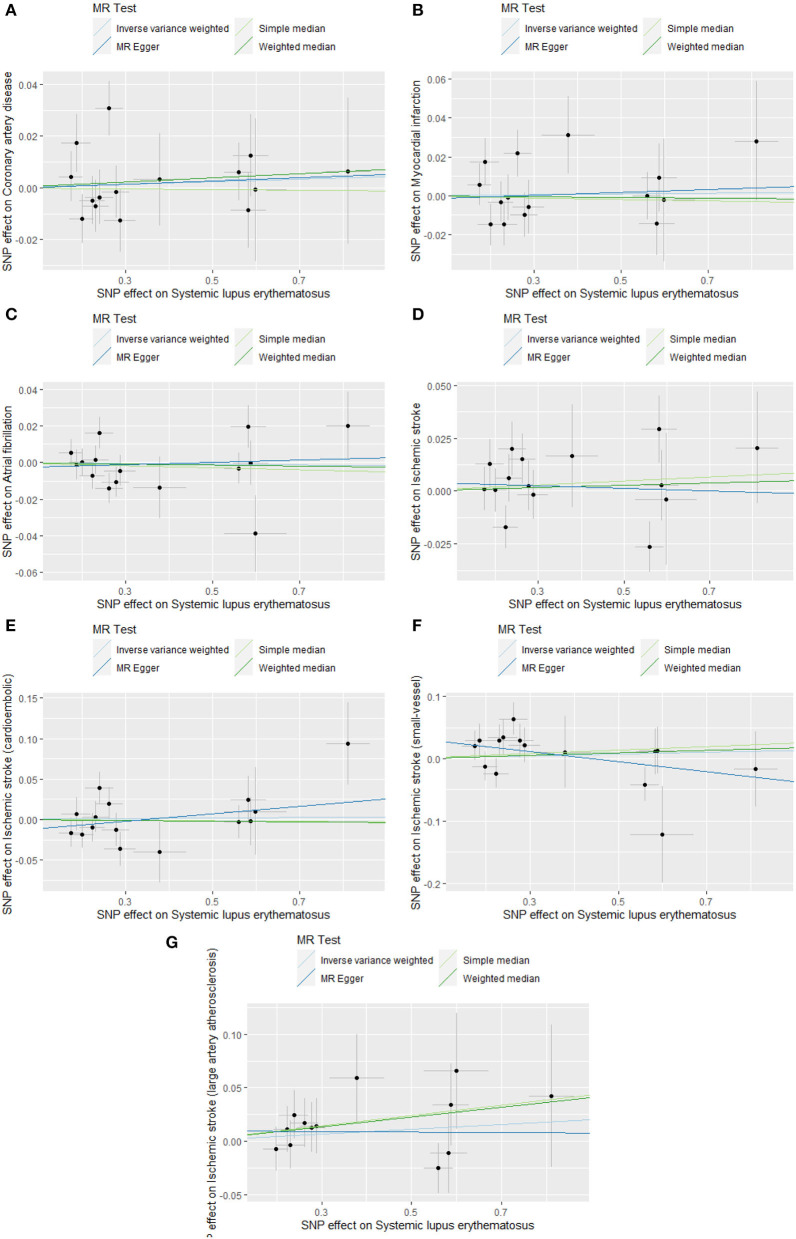
Scatter plots of causal associations of SLE on CVDs. SLE, systemic lupus erythematosus; CVDs, cardiovascular diseases. **(A)** coronary artery disease; **(B)** myocardial infarction; **(C)** atrial fibrillation; **(D)** ischemic stroke; **(E)** cardioembolic stroke; **(F)** small vessel stroke; **(G)** large artery stroke.

Cochran's *Q*-test revealed no significant heterogeneity among 15 SNPs (CAD: *p*-value = 0.327, MI: *p*-value = 0.399; AF: *p*-value = 0.170; IS: *p*-value = 0.218; CES: *p*-value = 0.144; SVS: *p*-value = 0.194; LAS: *p*-value = 0.052) (Table 1). Therefore, we chose the fixed-effects IVW method, indicating that there was no evidence to support the causal association between SLE and the risk of CVDs (CAD: OR 1.005, 95% CI 0.986–1.024, *p*-value = 0.619; MI: OR 1.002, 95% CI 0.982–1.023, *p*-value = 0.854; AF: OR 0.998, 95% CI 0.982–1.014, *p*-value = 0.795; IS: OR 1.006, 95% CI 0.984–1.028, *p*-value = 0.621; CES: OR 0.992, 95% CI 0.949–1.036, *p*-value = 0.707; SVS: OR 1.014, 95% CI 0.964–1.067, *p*-value = 0.589; LAS: OR 1.030, 95% CI 0.968–1.096, *p*-value = 0.352) (Table 2; Figure 3; Supplementary Figure 1). The effect estimates of the simple- and weighted-median method, MR-Egger, and MR-PRESSO were parallel to that of the IVW method (Table 2).

**Table 1:** Heterogeneity tests and MR-Egger intercept test of SLE for CVDs.

	Cochran's ***Q***-test				MR-Egger intercept test		
	**Q**	**Q_df**	***I*** **^2^**	***p*** **-value**	**Intercept**	**SE**	***p*** **-value**
Coronary artery disease	15.780	14	11.281	0.327	0.000	0.008	0.951
Myocardial infarction	14.698	14	4.749	0.399	−0.002	0.008	0.806
Atrial fibrillation	18.032	14	22.359	0.170	−0.003	0.006	0.624
Ischemic stroke	17.760	14	21.172	0.218	0.004	0.009	0.620
Cardioembolic stroke	19.576	14	28.482	0.144	−0.016	0.014	0.270
Small vessel stroke	18.298	14	23.490	0.194	0.036	0.018	0.064
Large artery stroke	23.512	14	40.455	0.052	−0.013	0.024	0.608

SLE, systemic lupus erythematosus; CVDs, cardiovascular diseases; SE, standard error.

**Table 2:** MR estimates of a causal association between SLE and CVDs.

**Outcome (CVDs)**	**MR method**	**NO. of SNPs**	**OR**	**95%CI**	***p*** **-value**
Coronary artery disease	IVW	15	1.005	0.986–1.024	0.619
	Simple median	15	0.999	0.974–1.025	0.926
	Weighted median	15	1.008	0.985–1.032	0.501
	MR Egger	15	1.006	0.962–1.053	0.794
	MR-PRESSO	15	1.005	0.986–1.024	0.627
Myocardial infarction	IVW	15	1.002	0.982–1.023	0.854
	Simple median	15	0.996	0.967–1.027	0.817
	Weighted median	15	0.998	0.971–1.027	0.904
	MR Egger	15	1.007	0.960–1.057	0.766
	MR-PRESSO	15	1.002	0.982–1.023	0.857
Atrial fibrillation	IVW	15	0.998	0.982–1.014	0.795
	Simple median	15	0.994	0.973–1.017	0.620
	Weighted median	15	0.997	0.977–1.018	0.799
	MR Egger	15	1.006	0.971–1.043	0.742
	MR-PRESSO	15	0.998	0.982–1.014	0.798
Ischemic stroke	IVW	15	1.006	0.984–1.028	0.621
	Simple median	15	1.009	0.978–1.041	0.557
	Weighted median	15	1.006	0.976–1.036	0.716
	MR Egger	15	0.994	0.945–1.045	0.815
	MR-PRESSO	15	1.006	0.984–1.028	0.629
Cardioembolic stroke	IVW	15	0.992	0.949–1.036	0.707
	Simple median	15	0.983	0.926–1.044	0.575
	Weighted median	15	0.989	0.938–1.043	0.689
	MR Egger	15	1.050	0.953–1.156	0.341
	MR-PRESSO	15	0.992	0.949–1.036	0.712
Small vessel stroke	IVW	15	1.014	0.964–1.067	0.589
	Simple median	15	1.028	0.956–1.106	0.454
	Weighted median	15	1.020	0.953–1.092	0.566
	MR Egger	15	0.922	0.832–1.022	0.147
	MR-PRESSO	15	1.014	0.964–1.067	0.597
Large artery stroke	IVW	15	1.030	0.968–1.096	0.352
	Simple median	15	1.027	0.957–1.103	0.459
	Weighted median	15	1.016	0.951–1.085	0.638
	MR Egger	15	1.066	0.924–1.231	0.398
	MR-PRESSO	15	1.030	0.968–1.096	0.368

SLE, systemic lupus erythematosus; CVDs, cardiovascular diseases; OR, odds ratio; CI, confidence interval, IVW, inverse-variance weighted method; MR-PRESSO, Mendelian randomization pleiotropy residual sum and outlier test.

To reduce the bias resulting from horizontal pleiotropy, we performed a MR-Egger intercept test and leave-one-out analysis. The result of MR-Egger intercept test did not reveal directional pleiotropy (CAD: intercept = 0.000, *p*-value = 0.951; MI: intercept = −0.002, *p*-value = 0.806; AF: intercept = −0.003, *p*-value = 0.624; IS: intercept = 0.004, *p*-value = 0.620; CES: intercept = −0.016, *p*-value= 0.270; SVS: intercept = 0.036, *p*-value = 0.064; LAS: intercept = −0.013, *p*-value = 0.608) (Table 1). Meanwhile, the leave-one-out analysis also demonstrated the robustness of the MR effect estimates (Supplementary Figure 2). Based on the sample size of the CVDs GWAS meta-analysis, there was >80% power to detect the associations of SLE with the risk of CAD, MI, AF, IS, and its subtypes for effect size (OR) of ~0.9 (Supplementary Table 3).

## Discussion

In the study, we systematically evaluated the causal association between SLE and the risk of CVDs by a two-sample MR analysis. We did not provide the causal evidence that genetically predicted SLE could increase the risk of CVDs (CAD, MI, AF, IS, CES, SVS, and LAS). The sensitivity analyses also displayed that horizontal pleiotropy was absent, revealing the robustness of effect estimates.

Previous multiple observational studies have proclaimed that SLE was positively associated with the incidence of CVDs. A 10-year follow-up cohort study performed by Yafasova et al. (32) found that patients with SLE had a higher risk of AF, IS, and MI. In line with the above study, another meta-analysis also suggested that the incidence of cardiovascular events was 25.4% among patients with SLE, including acute MI (4.1%) and stroke (7.3%) (33).

However, other studies revealed that the prevalence rate of coronary heart disease (CHD) and IS did not appear differently between patients with SLE and age- and gender-matched controls (34, 35). Our MR analysis also did not provide sufficient evidence that genetically predicted SLE was in connection with the increased risk of CVDs. Given the random allocation of genetic variants, MR analysis could provide more reliable results, compared with the observational study. Previous positive results might be triggered by several common confounders shared by SLE and CVDs. Obesity is a well-acknowledged risk factor for CVDs (36, 37), and it is also related to worse disease activity and higher levels of inflammation markers for SLE (37). Therefore, obesity is an extremely important common risk factor for SLE and CVDs. Apart from obesity, cigarette consumption also tended to act as another common risk (38, 39). In addition, long-term use of glucocorticoids, the fundamental drug for SLE, could lead to glucose-lipid metabolism disturbance to further induce the development of CHD, IS, and MI (40, 41).

The main strength of this study was that we estimated the causal relationship of SLE with CAD, MI, AF, IS, and its subtypes by two-sample MR analysis for the first time. In addition, we largely overcame the interference of potential confounders. However, there were still several limitations to this study. First, studies indicated disease activity was positively associated with CVDs among patients with SLE (42, 43). However, disease activity was not reported in the original SLE GWAS, which could have contributed to the definition of SLE status (active or remission), so we could not explore whether higher disease activity of SLE increased the rate of cardiovascular events. Second, the incidence of CVDs was different in different racial patients with SLE (44). As ~15.8% of AF and 23% of CAD were participants without European ancestry, our MR study might be slightly influenced by population effects. In the end, based on our MR results, although the ORs of AF and CES were <1 and others were more than 1, all the *p*-values were considerably >0.05. This inconsistency may be due to the insufficient sample size. Therefore, it is necessary to conduct SLE GWAS with a larger sample size to more demonstrate whether there is a causality between SLE and CVDs.

## Conclusion

Overall, our research provided no evidence that genetically predicted SLE was causally associated with the risk of CVDs by a two-sample MR analysis.

## Data availability statement

The original contributions presented in the study are included in the article/Supplementary material, further inquiries can be directed to the corresponding author/s.

## Ethics statement

Ethical review and approval was not required for the study on human participants in accordance with the local legislation and institutional requirements. Written informed consent for participation was not required for this study in accordance with the national legislation and the institutional requirements.

## Author contributions

JB and YF provided the idea, designed this study protocol, and revised the manuscript. SH and FH collected and analyzed data. CM and FT were responsible for the drawing. SH, FH, and CM contributed to the manuscript writing. All authors read and consented to the final manuscript.

## Funding

This study was supported by the National Natural Science Foundation of China (Grant No. 81803980) and the Research Project of Zhejiang Chinese Medical University (Grant Nos. 2019ZG22 and 2021JKZKTS012B).

## Conflict of interest

The authors declare that the research was conducted in the absence of any commercial or financial relationships that could be construed as a potential conflict of interest.

## Publisher's note

All claims expressed in this article are solely those of the authors and do not necessarily represent those of their affiliated organizations, or those of the publisher, the editors and the reviewers. Any product that may be evaluated in this article, or claim that may be made by its manufacturer, is not guaranteed or endorsed by the publisher.
