# Supplementary material for: Systemic lupus erythematosus and the risk of cardiovascular diseases: A two-sample Mendelian randomization study
Source: Front Cardiovasc Med. 2022 Sep 2;9:896499. doi: 10.3389/fcvm.2022.896499 (PMC9478435; doi:10.3389/fcvm.2022.896499)
Supplement: Supplementary file 1 [file Data_Sheet_1.docx]

SUPPLEMENTAL MATERIALS

Supplemental Table 1. Description of data sources about the MR analyses.

Supplemental Table 2. Detailed information for the genetic variants associated with SLE.

Supplemental Table 3. Power calculations for the Mendelian randomization analysis.

Supplemental Figure 1. Forest plots of SNPs associated with systemic lupus erythematosus (SLE) and cardiovascular diseases (CVDs).

Supplemental Figure 2. Leave-one-out analysis for systemic lupus erythematosus (SLE) and cardiovascular diseases (CVDs).

Supplemental Table 1. Description of data sources about the MR analyses.

| Trait | First Author (Year) | Sample size (Cases/Controls) | Population |
| --- | --- | --- | --- |
| SLE | Bentham J et al (2015) | 4,036/6,959 | European |
| CAD/MI | Nikpay M et al (2015) | 60,801/123,504 (43,676/128,199) | Mix (77% European) |
| AF | Roselli C et al (2018) | 65,446/522,744 | Mix (84.2% European) |
| IS | Malik R et al (2018) | 34,217/406,111 | European |
| CES | Malik R et al (2018) | 7,193/406,111 | European |
| SVS | Malik R et al (2018) | 5,386/406,111 | European |
| LAS | Malik R et al (2018) | 4,373/406,111 | European |

MR, Mendelian Randomization; SLE, Systemic Lupus Erythematosus; CAD, Coronary Artery Disease; MI, Myocardial Infarction; AF, Atrial Fibrillation; IS, Ischemic Stroke; CES, Cardioembolic Stroke; SVS, Small Vessel Stroke; LAS, Large Artery Stroke.

Supplemental Table 2. Detailed information for the genetic variants associated with SLE.

| SNP | Locus | Chr | Pos | EA | OA | MAF | Beta | SE | *p*-value | R^2^ | *F*-statistic |
| --- | --- | --- | --- | --- | --- | --- | --- | --- | --- | --- | --- |
| rs10028805 | *BANK1* | 4 | 102737250 | A | G | 0.664 | -0.174 | 0.028 | 4.50E-10 | 0.014 | 151.162 |
| rs10036748 | *TNIP1* | 5 | 150458146 | T | C | 0.312 | 0.278 | 0.032 | 2.83E-18 | 0.033 | 376.221 |
| rs10488631 | *IRF5* | 7 | 128594183 | C | T | 0.173 | 0.582 | 0.041 | 2.66E-44 | 0.097 | 1180.799 |
| rs11644034 | *IRF8* | 16 | 85972612 | A | G | 0.837 | -0.288 | 0.036 | 1.25E-15 | 0.023 | 253.983 |
| rs11889341 | *STAT4* | 2 | 191943742 | T | C | 0.324 | 0.560 | 0.033 | 1.17E-65 | 0.137 | 1747.832 |
| rs17849501 | *SMG7* | 1 | 183542323 | T | C | 0.123 | 0.811 | 0.050 | 1.63E-59 | 0.142 | 1817.466 |
| rs2431697 | *MIR146A* | 5 | 159879978 | C | T | 0.610 | -0.223 | 0.029 | 3.23E-14 | 0.024 | 266.762 |
| rs2736340 | *BLK* | 8 | 11343973 | T | C | 0.303 | 0.262 | 0.032 | 2.14E-16 | 0.029 | 329.188 |
| rs34572943 | *ITGAM* | 16 | 31272353 | A | G | 0.199 | 0.588 | 0.041 | 1.74E-47 | 0.110 | 1360.666 |
| rs6568431 | *PRDM1* | 6 | 106588806 | C | A | 0.578 | -0.199 | 0.029 | 4.33E-12 | 0.019 | 216.223 |
| rs6740462 | *SPRED2* | 2 | 65667272 | A | C | 0.231 | 0.186 | 0.033 | 2.31E-08 | 0.012 | 137.290 |
| rs6932056 | *TNFAIP3* | 6 | 138242437 | C | T | 0.054 | 0.599 | 0.072 | 1.23E-16 | 0.037 | 418.078 |
| rs704840 | *TNFSF4* | 1 | 173226195 | G | T | 0.328 | 0.231 | 0.031 | 1.65E-13 | 0.024 | 265.083 |
| rs7444 | *UBE2L3* | 22 | 21976934 | C | T | 0.247 | 0.239 | 0.033 | 1.30E-13 | 0.021 | 238.685 |
| rs7726414 | *TCF7* | 5 | 133431834 | T | C | 0.071 | 0.378 | 0.061 | 9.17E-10 | 0.019 | 211.684 |

SNP, single-nucleotide polymorphism; Chr, chromosome; Pos, position; EA, effect allele; OA, other allele, MAF: Minor allele frequency; SE: standard error; R^2^, the proportion of variance explained by the SNP.

R^2^ = 2×MAF×(1–MAF)×Beta^2^, where MAF is the minor allele frequency and beta is the effect of the SNP on SLE.

*F*-statistic = R^2^ ×(n-2)/(1-R^2^), where n stands for the sample size.

Supplemental Table 3. Power calculations for the Mendelian randomization analysis.

| Outcome | Sample size | Proportion of cases | Odds ratio estimated for α^1^ |
| --- | --- | --- | --- |
| Coronary Artery Disease | 184,305 | 0.330 | 0.984 |
| Myocardial Infarction | 171,875 | 0.254 | 0.982 |
| Atrial Fibrillation | 588,210 | 0.111 | 0.987 |
| Ischemic Stroke | 440,328 | 0.078 | 0.982 |
| Cardioembolic Stroke | 413,304 | 0.017 | 0.961 |
| Small Vessel Stroke | 411,497 | 0.013 | 0.955 |
| Large Artery Stroke | 410,484 | 0.011 | 0.951 |

Note: ^1^ Odds ratio estimated for α, the smallest effect detected by the sample size to provide 80% statistical power at an alpha level of 5%.

Supplemental Figure 1. Forest plots of SNPs associated with systemic lupus erythematosus (SLE) and cardiovascular diseases (CVDs).


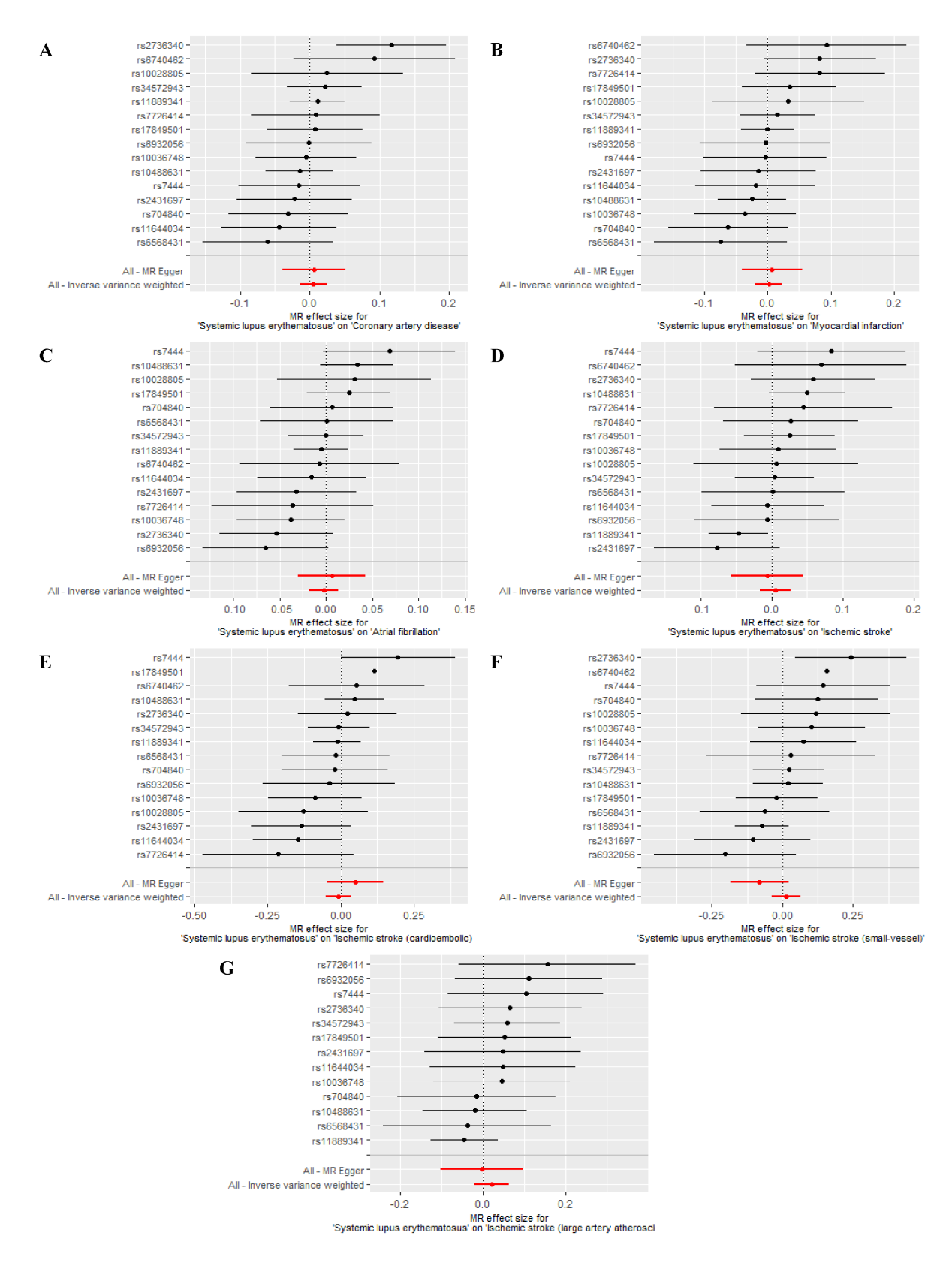


A, Coronary Artery Disease; B, Myocardial Infarction; C, Atrial Fibrillation; D, Ischemic Stroke; E, Cardioembolic Stroke; F, Small Vessel Stroke; G, Large Artery Stroke.

Supplemental Figure 2. Leave-one-out analysis for systemic lupus erythematosus (SLE) and cardiovascular diseases (CVDs).


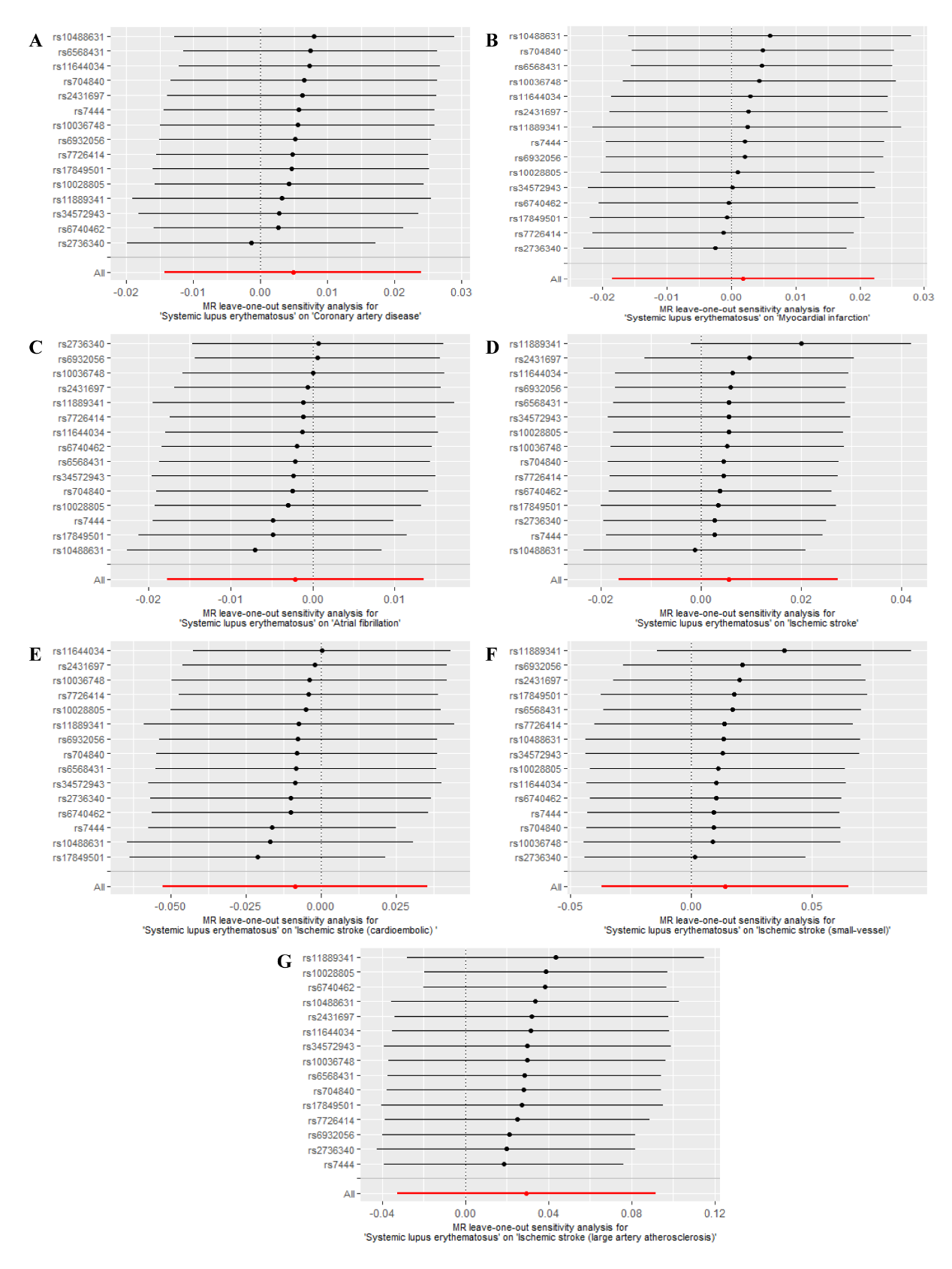


A, Coronary Artery Disease; B, Myocardial Infarction; C, Atrial Fibrillation; D, Ischemic Stroke; E, Cardioembolic Stroke; F, Small Vessel Stroke; G, Large Artery Stroke
